# Pulmonary Crohn's Disease Masquerading as Lymphoma

**DOI:** 10.14309/crj.0000000000001247

**Published:** 2024-01-04

**Authors:** William Beaty, Anila Katragadda, Rany Condos, Bari Dane, Suparna Sarkar, Emily Shaffer, Shannon Chang

**Affiliations:** 1 New York University Grossman School of Medicine, New York, NY; 2 Kansas City University College of Osteopathic Medicine Kansas City, MO; 3 Department of Medicine, New York University Grossman School of Medicine, New York, NY; 4 Department of Radiology, New York University Grossman School of Medicine, New York, NY; 5 Department of Pathology, New York University Grossman School of Medicine, New York, NY

**Keywords:** Crohn's disease, inflammatory bowel disease, extraintestinal manifestations

## Abstract

Although extraintestinal manifestations of inflammatory bowel disease (IBD) are common, pulmonary IBD is extremely rare. Owing to its nonspecific clinical, radiologic, and pathologic features, pulmonary IBD is difficult to diagnose and may mimic more concerning disease processes. We present a rare case of a patient with known Crohn's disease whose initial presentation was highly suspicious for malignancy before further investigation revealed pulmonary IBD.

## INTRODUCTION

Extraintestinal manifestations (EIMs) of inflammatory bowel disease (IBD) are common; however, pulmonary IBD is extremely rare.^1–3^ IBD may affect all parts of the respiratory tract, causing airway disease, interstitial lung disease (ILD), and granulomatous lung disease (GLD).^4,5^ ILD is associated with ulcerative colitis, whereas GLD is associated with Crohn's disease (CD).^6^ The etiology, prevalence, and prognosis of pulmonary IBD are poorly understood.

We present a case of a patient with CD who presented with dyspnea and constitutional symptoms found to have lung nodules and a mediastinal mass initially highly concerning for lymphoma; surgical biopsy revealed granulomatous inflammation consistent with a rare presentation of pulmonary IBD.

## CASE REPORT

A 23-year-old woman with CD presented to the hospital with subacute symptoms of shortness of breath, chest discomfort, fatigue, and generalized weakness.

Her history with Crohn's colitis began at the age of 15 years when she developed abdominal pain and weight loss; 2 years later, she developed a vaginal fistula and anal strictures requiring dilatation. She was started on infliximab with resolution of IBD symptoms but developed drug-induced psoriasis requiring methotrexate initiation. Four months before presentation, she was transitioned from infliximab to risankizumab because of ongoing psoriasis. Her abdominal symptoms remained quiescent in this period.

One month before presentation, the patient developed persistent cough and congestion. During this time, she developed an erythematous, painful nodule on her right leg consistent with erythema nodosum. One week before presentation, she developed full-body soreness with severe fatigue and exertional dyspnea, after which she decided to present to the emergency department. She continued using risankizumab during this period.

**Figure 1.**
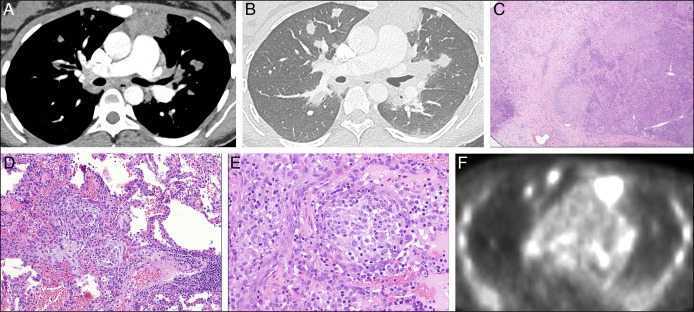
(A) CT angiogram (soft tissue window) and (B) CT angiogram (lung window) showing anterior mediastinal mass, hilar adenopathy, bilateral lung nodules. (C–E) VATS pathology specimen showing areas of non-necrotizing and poorly-formed necrotizing granulomas without evidence of malignancy. (F) PET-CT showing FDG-avid anterior mediastinal mass and diffuse mildly FDG-avid nodules throughout lung parenchyma.

At presentation, she described dyspnea and pleuritic chest pain. She also noted weight loss and night sweats and denied nausea, vomiting, diarrhea, constipation, and abdominal pain. Her initial vitals were notable for afebrile, tachycardia to 110 bpm (normal 60–100), and 99% SpO_2_ on room air. Initial laboratory test results were notable for white blood cell count 12.8 × 10^3^ cells/μL (normal 4.0–10.0) with 79% neutrophils, hemoglobin 12.0 g/dL (normal 11.2–15.7), D-Dimer 1231 ng/mL (normal <230), and erythrocyte sedimentation rate 120 mm/hr (normal 0–20). Computed tomography (CT) showed an anterior mediastinal mass with soft-tissue nodules throughout the lungs with bilateral perihilar adenopathy, suspicious for diffuse metastatic disease (Figure 1). There was no imaging evidence of neoplasm in the abdomen or pelvis. Her clinical picture was believed to be most concerning for Hodgkin lymphoma vs primary mediastinal B-cell lymphoma in the setting of previous infliximab use.

IR-guided biopsy of the mediastinal mass showed only necrotizing granulomatous inflammation. This was considered nondiagnostic, given the lower yield of such a biopsy, and 2 weeks later, the patient successfully underwent video-assisted thoracic surgery left upper and lower lobe wedge resections. Pathology demonstrated areas of non-necrotizing and poorly formed necrotizing granulomas without evidence of malignancy (Figure 1). The specimen was also cultured for bacterial, mycobacterium, and fungal specimens without growth.

She underwent PET-CT, which showed a large fluorodeoxyglucose-avid anterior mediastinal mass and diffuse mildly fluorodeoxyglucose-avid nodules throughout the lung parenchyma (Figure 1). Her laboratory test results were also notable for a normal angiotensin-converting enzyme level and negative QuantiFERON. Throughout this period, she continued receiving risankizumab. She did not receive steroids or any other IBD-directed therapies.

Over the following months, the patient's symptoms and inflammatory markers gradually resolved without changes in medications. She noted feeling at her best in the days after risankizumab injections. Three months after presentation, she received a noncontrast chest CT that revealed improvement in parenchymal nodules and decreased lymphadenopathy. Six months after presentation, she feels well and leads an active lifestyle with occasional diarrhea at her previous baseline.

Based on the time course of her symptoms, it was considered most likely that the patient's clinical course represented primary Crohn's GLD unmasked by cessation of infliximab. Drug reaction to risankizumab was also considered, but less likely based on the resolution of symptoms without drug discontinuation as well as the absence of any known association. Sarcoidosis was also considered less likely because of the presence of focal necrotizing granulomas in addition to the normal angiotensin-converting enzyme level.

## DISCUSSION

We present a case of a patient with pulmonary manifestations of CD whose initial disease presentation was highly convincing for malignancy and required multiple invasive tests to confidently exclude lymphoma.

EIMs of IBD are common, affecting 50% of patients.^5^ The most commonly affect organ systems are the musculoskeletal system, skin, eyes, and hepatobiliary tract.^1^ Pulmonary manifestations, however, are rare with an estimated prevalence of 0.4%.^5^ There is high heterogeneity in patient presentations, and all segments of the bronchopulmonary tract may be affected.^2,4,6^ Symptoms often present during gastrointestinal disease flares; however, in some cases, such as in this patient, isolated pulmonary disease occurs. ILD is more strongly associated with UC, whereas GLD is more associated with CD.^6^ In a recent database review by Eliadou et al,^6^ 68% of pulmonary IBD patients were male with a mean age of 47 years. Interestingly, only 36% of patients reported the presence of other EIMs. In GLD patients, the most common presenting symptoms were cough (64%) and dyspnea (29%); fever and lethargy were also reported. Pulmonary EIMs may be drug-induced.^3,5^ Mesalamine and sulfasalazine are the most implicated drugs; however, biologics have been reported as well.^5^

Diagnosing pulmonary IBD is difficult because the clinical, radiologic, and pathologic findings are nonspecific. This is compounded by the rarity of pulmonary IBD compared with mimics, such as lymphoma and sarcoidosis. Indeed, in this case, we cannot completely rule out a risankizumab reaction, unmasking of CD after anti–tumor necrosis factor withdrawal, or a new diagnosis of sarcoidosis. Fortunately, pulmonary EIMs carry a favorable prognosis because patients typically respond very well to steroid therapy,^1,3,7^ although in some cases, additional immunosuppression is necessary.

## DISCLOSURES

Author contributions: W. Beaty was primarily responsible for manuscript drafting. A. Katragadda assisted in manuscript drafting. R. Condos, B. Dane, S. Sarkar, and E. Shaffer assisted in procuring images and in editing the manuscript. S. Chang was principally responsible for oversight of the project and editing the manuscript and is the article guarantor.

Financial disclosure: None to report.

Informed consent was obtained for this case report.
